# How lactate affects immune strategies in lymphoma

**DOI:** 10.3389/fmolb.2024.1480884

**Published:** 2024-10-11

**Authors:** Yuehan Zhou, Jinzhan Lou, Yuqin Tian, Jinlei Ding, Xiaobo Wang, Bo Tang

**Affiliations:** ^1^ Department of Hematology, The Second Affiliated Hospital of Dalian Medical University, Dalian, China; ^2^ Department of Thoracic Surgery, The Second Affiliated Hospital of Dalian Medical University, Dalian, China

**Keywords:** lactate, lymphoma, immune cells, immune checkpoints, HIF-1α, MYC, MTOR signaling, NF-κB signaling

## Abstract

Tumor cells undergo metabolic reprogramming through shared pathways, resulting in a hypoxic, acidic, and highly permeable internal tumor microenvironment (TME). Lactate, once only regarded as a waste product of glycolysis, has an inseparable dual role with tumor immunity. It can not only provide a carbon source for immune cells to enhance immunity but also help the immune escape through a variety of ways. Lymphoma also depends on the proliferation signal of TME. This review focuses on the dynamic process of lactate metabolism and immune function changes in lymphoma and aims to comprehensively summarize and explore which genes, transcription factors, and pathways affect the biological changes and functions of immune cells. To deeply understand the complex and multifaceted role of lactate metabolism and immunity in lymphoma, the combination of lactate targeted therapy and classical immunotherapy will be a promising development direction in the future.

## 1 Introduction

The terms “cancer metabolism” and “metabolic reprogramming” are frequently used to describe a set of shared pathways observed in highly proliferating tumors or cancer cells (Faubert et al., 2020). Tumor cells reprogram their metabolic pathways to meet the bioenergetic, biosynthetic, and redox demands for rapid tumor cell proliferation, resulting in hypoxia, nutrient deficiencies, and elevated levels of metabolic byproducts in the tumor microenvironment (TME) (Sahai et al., 2020). The predominance of active glycolysis over aerobic glucose metabolism in this case leads to elevated lactate, thus becoming one of the most important reasons for the composition of the microenvironment. Lactate has long been neglected in the exploration of human tumors, considered only as a waste product produced by glycolysis, with only a role as a biomarker of malignancy. Recently, in TME, lactate is no longer treated as a waste produced by cellular metabolism, but as a powerful signaling molecule that influences the behavior of tumor cells and surrounding cells (Brooks, 2009; Rabinowitz and Enerbäck, 2020). The understanding of lactate has been gradually improved and enriched. First, lactate re-establishes metabolic coupling either between cancer cells or between cancer and non-malignant cells to power and sustain tumor growth. Second, in recent years, the study of the effects of lactate on tumor cells has been extended to the field of epigenetics, and the discovery that accumulated lactate is converted to lactyl coenzyme A via lactylation-regulated genes has become a research hotspot in a single leap (Ippolito et al., 2019; Zhang et al., 2019). Finally, lactate can help tumor cells better adapt to TME and avoid immune attack by inhibiting immune surveillance mediated by immune cells (Ngwa et al., 2019). A study reported that lactate can serve as a carbon source for mammalian cells to utilize (Brooks, 2009), and this report provides a new perspective on lactate-mediated interactions between tumor cells and immune cells: lactate has a dual role, not only promoting immune evasion, but also seems able to provide a carbon source for immune cells to aid tumor immunity. The latest report also emphasizes the viewpoint: lactate related lactylation affects gene expression in tumor cells and immune cells, leading to immune suppression, tumor progression, and poor prognosis (Zhang et al., 2024).

The role of the microenvironment in lymphomas has historically been underestimated. However, recent studies have demonstrated that, in most cases, diffuse large B-cell lymphoma (DLBCL) depends on the proliferative signals of TME to grow and achieve escape from immune surveillance. Similar to other tumors, lymphoma produces large amounts of lactate. Consequently, TME is not only a significant factor in the pathogenesis and prognosis of lymphoma but also the foundation of therapeutic strategies and drug resistance (Cycon et al., 2009; Scott and Gascoyne, 2014). Lactate metabolism can not only directly affect the immune cells to regulate the immune function of lymphoma, but also indirectly affect the immune cells by affecting genes and immune checkpoints, thus making the lymphoma cells immune escape. Lactate metabolism and immune escape in lymphoma are a dynamic process associated with lymphoma initiation and progression. How lactate has affected immunity in lymphoma is well worth exploring.

The latest advances in single-cell technology, such as single-cell RNA sequencing (scRNA-Seq) and time-of-flight cytometry (CyTOF), in the molecular subtype classification of myeloid cells in Glioblastoma (GBM), macrophages are divided into two clusters with different functional states: immunosuppressive cells and proliferating macrophages. A new functional state of macrophages and microglia has been identified in human GBM tumors, where macrophages exhibit upregulation of immunosuppressive cytokines and activate the tricarboxylic acid cycle. Artificial intelligence accurately characterizes tumor-associated macrophages (TAM) and identifies specific tumor regulatory functions in GBM, which can promote the understanding of TAM heterogeneity in GBM (Khan et al., 2023).

In this review, we review and describe the metabolic pivotal role of lactate in lymphoma. In addition, we focused on exploring the crosstalk between lactate and immune function in lymphoma. The regulation of immune function includes the immune cells in TME, immune checkpoints and dysregulation of lactate-related transcription factors and signaling pathways such as mammalian target of rapamycin (mTOR), and hypoxia-inducible factor (HIF). We also discuss how we can influence the biological changes and functions of lymphma and immune cells through specific genes, transcription factors, and pathways, and consequently regulate immune responses.

## 2 High lactate in lymphoma

Otto Heinrich Warburg first described the Warburg effect of tumors in the early 1920s (Warburg, 1956). Even in sufficient oxygen, tumor cells take up glucose about ten times faster than normal tissues and metabolize large amounts of lactate over a given period (Levine and Puzio-Kuter, 2010). Lymphoma cells also have a Warburg effect, namely, “aerobic glycolysis”. Although glycolysis can only produce two adenosine triphosphate (ATP) molecules per glucose molecule, it also produces two lactic acids. Compared with oxidative phosphorylation (OXPHOS), the latter produces 36 ATP molecules per glucose molecule. However, due to the high metabolic rate of aerobic glycolysis and high glucose intake, this process produces lactate far beyond normal tissues (Levine and Puzio-Kuter, 2010). This high glucose uptake is associated with a poor prognosis in DLBCL (Cho et al., 2015), and is one of the characteristics of aggressive lymphomas. The preferential production of lactate leads to the accumulation of lactate in the TME, which in turn leads to lactic acidosis. Lactic acidosis is pathophysiologically classified into type A and type B. Type B occurs mainly in hematologic malignancies, especially induced lymphomas producing lactic acidosis. It is considered a tumor emergency, a life-threatening emergency that leads to high mortality and poor outcomes (Wang et al., 2022; Hamada et al., 2020; Duriseti et al., 2021; McKay et al., 2017; Friedenberg et al., 2007; Sillos et al., 2001). Case reports also confirm the view (Soleja et al., 2016). More importantly, under normal conditions, when lactate accumulates it can serve to drive appropriate physiological responses, but the activity is reversed in the case of cancer, instead suppressing the anti-cancer immune response and promoting immune escape from the tumor (Rabinowitz and Enerbäck, 2020).

## 3 Two important proteins in lactate metabolism

**FIGURE 1:**
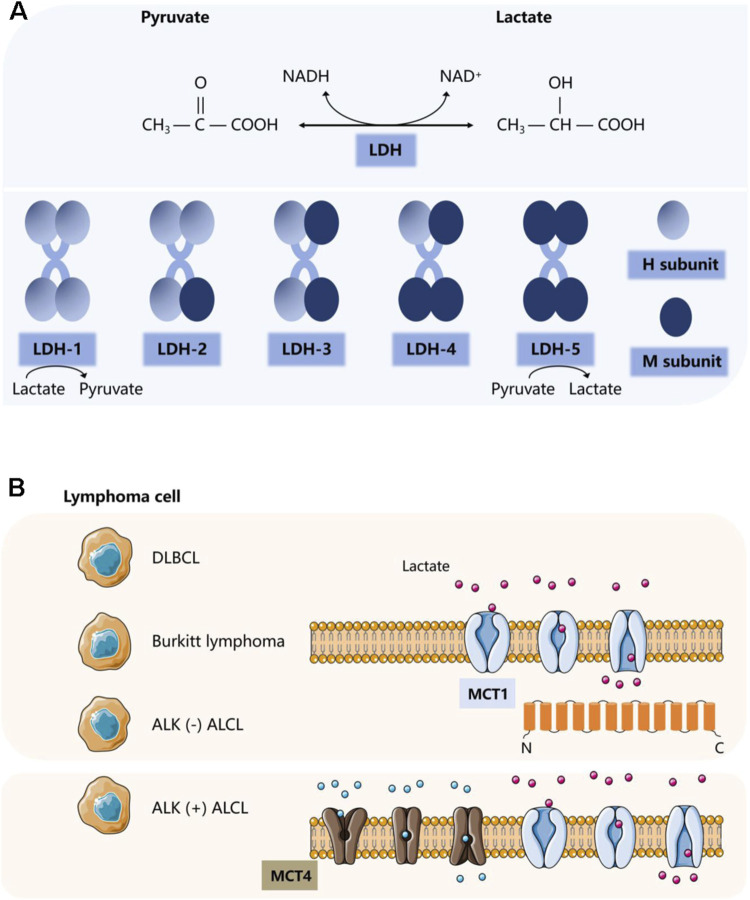
Two important proteins involved in lactate metabolism. **(A)** Classification and corresponding functions of LDH. **(B)** Classification and corresponding functions of MCT.

The production of lactate and its rapid transport depends on several enzymes and proteins. In tumors, upregulation and sustained activation of hypoxia-inducible factor-1α (HIF-1α) and c-Myc lead to aberrant expression of several glycolytic enzymes and monocarboxylate transporter (MCT) proteins, including lactate dehydrogenase A (LDHA), MCT1 and MCT4 (Gordan et al., 2007; Masoud and Li, 2015). In lymphoma, there is evidence of the activation of HIF-1α and c-Myc (Evens et al., 2010; Ott et al., 2013), as well as the activation of LDHA, MCT1, and MCT4. These factors not only are activated but also influence various functions and prognostic markers in lymphomas (Zhao et al., 2023). Here, we will focus on lactate dehydrogenase (LDH) and MCT proteins (Figure 1).

### 3.1 Lactate dehydrogenase

LDH is present in blood cells and lymphoid tissues. It is not only often used as a biochemical indicator for diagnosis and therapeutic monitoring of lymphoma, but for many years it has been considered to correlate with a poor prognosis in lymphoma patients. As an independent prognostic marker, it is one of the most important prognostic determinants of the international prognostic index (Project, 1993). LDH, consisting of the M and H protein subunits encoded by the LDHA and LDHB genes, respectively, is a tetrameric enzyme essential for lactate synthesis that reversibly catalyzes the conversion of pyruvate to lactate or lactate to pyruvate. It is assembled in a tissue-dependent manner in five different heterotetramers or homotetramers to form five isozymes: LDH-1 (4H), LDH-2 (3H1M), LDH-3 (2H2M), LDH-4 (1H3M), and LDH-5 (4M). The LDHA isoforms are predominantly expressed in skeletal muscle, and LDH-5 (LDHA4) prevents pyruvate from entering the mitochondrial tricarboxylic acid cycle (Markert et al., 1975) and promotes the conversion of pyruvate to lactate. Whereas LDHB isoforms are usually located in the heart and brain, LDH-1 (LDHB4) plays the opposite role, preferentially converting lactate to pyruvate (Ždralević et al., 2018; Feng et al., 2018). LDHA and its downstream signals, as potential biomarkers, are positively correlated with macrophage density, such as in glioblastoma. Recent studies have shown that LDHA activates two transcriptional co-activators, yes-related protein 1 (YAP1) and transcriptional activator 3 (STAT3), in glioblastoma cells through a directed extracellular signal-regulated kinase (ERK) pathway. They can coordinate the upregulation of C-C motif chemokine ligand 2 (CCL2) and CCL7, thereby triggering macrophage infiltration into the TME. Conversely, the recruited macrophages produce extracellular vesicles that release LDHA. LDHA inhibitors can not only regulate the inhibitory anti-tumor immune function of macrophages but also inhibit the growth and development of tumors (Khan et al., 2024).

Lactate production is inextricably linked to the regulation of LDH. Elevated serum lactate in patients with lymphoma is largely progressive (Ruan et al., 2021). Elevated LDH levels in patients with aggressive lymphoma may be due to increased cell renewal and hypermetabolism of the tumor. In malignant lymphomas, the intracellular mitochondrial machinery is altered, and apoptosis is dysregulated, and tumor cells release intracellular enzymes through damaged cell membranes (Jurisic et al., 2015). It suggests that abnormally enhanced tumor metabolism and tumor cell necrosis cause increased release of LDH into the blood. The rapid proliferation of tumor cells results in a hypoxic environment as well as mutations in certain oncogenes and oncostatin genes, such as HIF-1α and c-Myc, which enhance pyruvate production by accelerating the two rate-limiting steps in glycolysis involving hexokinase 2 (HK2) and fructose-2,6-bisphosphate. They also inhibit pyruvate dehydrogenase (PDH) phosphorylation by inducing pyruvate dehydrogenase kinase 1 (PDK1), which in turn inhibits its mediated mitochondrial metabolic activity of pyruvate. Meanwhile, both can induce LDHA gene expression and inhibit LDHB gene expression, further enhancing LDH-5 activity and decreasing LDH-1 activity (Feng et al., 2018). It ultimately leads to the conversion of pyruvate to lactate to promote the glycolytic process, resulting in increased lactate production and conversion of NADH to NAD+.

### 3.2 Monocarboxylate transporter

The characteristic glycolytic process described above leads to the accumulation of lactate in the cytoplasmic lysate (Warburg, 1924; Warburg and Research, 1925). Consequently, to prevent intracellular acidification, lactate and H exocytosis must be transported to the extracellular space and also exchanged between cell populations (Certo et al., 2021).

MCTs are plasma membrane transport proteins essential for lactate shuttling. MCTs are highly expressed in stromal cells of DLBCL. In addition to the high rate of glycolysis exhibited by lymphoma cells that produce excess lactate, a large amount of lactate produced by the cell must be transported out of lymphoma cells through MCTs (Gooptu et al., 2017), mediating the secretion and reuptake of lactate or pyruvate, thus preventing cell death caused by acidosis. This process resulted in an increase in lactate levels in TME. MCTs belong to a class of transporter proteins encoded by a family of solute carrier proteins, which consists of 14 members (Singh et al., 2023). The most common cytosolic-localized proton-coupled transporter proteins of monocarboxylates are c-Myc-mediated MCT1 and HIF-1α-mediated MCT4. MCTs facilitate the transport of lactate and pyruvate in cancerous cells (e.g., (Halestrap and Wilson, 2012; Halestrap, 2012)). They also facilitate lactate shuttling between cancer cells and stromal cells in the TME. MCT1 is the most widely expressed and has a relatively high affinity for lactate. It acts as a transporter according to local lactate concentration gradients. In contrast, MCT4 acts as an efflux transporter, primarily in highly glycolytic tissues. The transmembrane auxiliary protein CD147 ensures that MCTs function as transporter proteins in the correct orientation at the cell surface (Ippolito et al., 2019). Emerging evidence suggests that proton-coupled lactate efflux from tumor cells or stromal cells contributes to remodeling the TME to maintain an acidic phenotype and promotes tumor spreading, leading to angiogenesis and invasive metastasis as well as immune escape (Kirk et al., 2000), which is associated with poor prognosis (Parks and Pouysségur, 2017).

MCT1 and MCT4 are differentially overexpressed in solid tumors in a variety of cancer types, such as lung, colon, and renal cancers (Ruan et al., 2017; Nakayama et al., 2012; Kim et al., 2015). Evidence also exists in hematologic malignancies such as myeloma and lymphoma (Walters et al., 2013). The significant differential expression of MCT in different lymphomas has been demonstrated in multiple experiments. In NHL, adverse clinical pathological features are significantly correlated with the expression of MCT1 (Afonso et al., 2019). Studies have confirmed that in DLBCL and Burkitt lymphoma, MCT1 is expressed at high levels in the absence of significant expression of MCT4 (Noble et al., 2017). Another study showed that in ALK (−) anaplastic large cell lymphoma (ALCL) tumor cells as well as B cells, natural killer/T cells, T cells, and classical Hodgkin lymphoma, only MCT1 is widely expressed. The expression of MCT4 is mainly localized to adjacent stromal cells. By comparison, only ALK (+) ALCL cells have high expression of MCT1 on the tumor cell membrane and widespread expression of MCT4 (Choi et al., 2022). Similarly, in T-cell lymphoma tissue, both MCT1 and MCT4 are overexpressed and associated with decreased OS and PFS, indicating poor prognosis (Zhao et al., 2023). Therefore, therapeutic strategies that disrupt lactate transport may be promising approaches for treating lymphoma (Le Floch et al., 2011; Parks et al., 2013; Doherty and Cleveland, 2013). AZD3965 (a first-of-its-kind MCT1 inhibitor) has been used in phase I clinical trials for high MCT1/low MCT4 cancers targeting this target (Halford et al., 2023). Its therapeutic effect may be related to inhibiting lipid biosynthesis and increasing tumor immune cell infiltration involving dendritic and natural killer (NK) cells (Beloueche-Babari et al., 2020).

## 4 Lymphoma pathogenic genes that are important promote lactate production

Dynamic regulation of lactate energy metabolism in cancer can be traced to a “trinity” of transcription factors: c-MYC, HIF-1, and p53 (Yeung et al., 2008).

### 4.1 HIF-1α

HIF-1 stimulates anaerobic glycolysis, which accumulates lactate and acidifies the TME, affecting cellular subpopulations in the TME including immune cells (Faubert et al., 2017). HIF-1α regulates the expression of genes encoding enzymes necessary for aerobic glycolysis (Darekar et al., 2012) and increases the production of metabolite lactate. For example, in myc-driven cell lines, HIF-1α and myc regulate the expression of HK2 and PDK1 (Mushtaq et al., 2015). The Warburg effect regulated by HIF-1α is observed in lymphoblastoid cells (Darekar et al., 2012; Mushtaq et al., 2015). During lactate metabolism, HIF-1α induces the overexpression of VEGF, which is involved in MCT protein 1-mediated lactate transport and subsequent inhibition of prolyl hydroxylase (PHD) (Horikawa et al., 2017; Rivera et al., 2015; Rivera and Bergers, 2015; Sonveaux et al., 2012). Inactivation of HIF PHDs will initiate transcription of target genes such as glucose transporter proteins, most glycolytic enzymes, MCT4, and VEGF (Semenza, 2003; Semenza, 2010). Finally, in solid tumors, HIF-1α-induced lactate promotes ferroptosis resistance in a pH-dependent manner, suggesting a promising therapeutic strategy (Yang et al., 2023). In the hypoxic microenvironment (HME) formed by malignant tumor cells, HIF-1α is more stable under the low pH condition of lactate formation and is not easy to be degraded, thus increasing its accumulation in cells (Zhao et al., 2024). HIF-1α regulates a variety of immune cells (such as T lymphocytes, macrophages, MDSCs) to modulate tumor immunity. In addition, it can also mediate the upregulation of PD-L1 and promote tumor immune escape (Noman et al., 2019; Deng et al., 2021). HIF-1α protein expression is also strongly dependent on some signaling pathways, such as PI3K/Akt/mTOR signaling pathway (Rashid et al., 2021).

HIF-1α is the best-studied isoform of the heterodimeric transcription factor HIF-1 and is normally expressed in human cells (Liu et al., 2020). Its overexpression is strongly associated with hematologic malignancies such as leukemia, lymphoma, and multiple myeloma (Zhao et al., 2024). Stabilization and upregulation of HIF-1α were observed in lymph node biopsies from both DLBCL and FL patients (Evens et al., 2008; Pangarsa et al., 2012). It can lead to overall translational repression as well as mitochondrial dysfunction during hypoxic stress in DLBCL and serves as one of the prognostic factors in assessing the likelihood of survival in DLBCL patients treated with R-CHOP (Evens et al., 2010). Furthermore, MYC and PI3K/AKT/mTOR independently increase HIF-1α expression (Pang et al., 2023). It has been demonstrated that HIF-1α may promote the viability and migration of activated B cell-like cells under hypoxia through the transcription of CXCR4 and activation of the AKT/mTOR pathway (Jin et al., 2023).

The immunosuppressive effect produced by HIF-1α is associated with the activation of several downstream effects. The HIF1α-VEGF signaling pathway described above regulates macrophage conversion to the M2 pro-angiogenic phenotype, thereby exerting a function in signaling (Colegio et al., 2014a; Kes et al., 2020). Moreover, lactate inhibits monocyte activation and dendritic cell differentiation by increasing HIF-1α stability (Colegio et al., 2014a; Gottfried et al., 2006; Nasi et al., 2013). As described above, lactate produced by activated DCs and other immune cells plays a role in regulating DCs by pathogenic autoimmune T cells through a HIF-1α-mediated mechanism (Sanmarco et al., 2023). This gives us new therapeutic ideas, but this mechanism remains to be further investigated and confirmed in hematologic malignant diseases.

### 4.2 MYC

The MYC oncogene plays a crucial role in a wide range of human solid tumors and various hematological malignancies, including B-cell and T-cell malignancies, especially infiltrative B-cell lymphomas such as DLBCLs and BLs (Pang et al., 2023; Aukema et al., 2011; Slack and Gascoyne, 2011), and it is considered to be a major regulator of cellular metabolism and proliferation (Dang, 2012). In lymphomas, MYC activation occurs through mutation, amplification, translocation, and various other molecular processes (Ott et al., 2013; Ruzinova et al., 2010; Leucci et al., 2008). MYC proteins, which are synergistic regulators of the Warburg effect with HIF-1α (Gordan et al., 2007), upregulate lactate production through multiple mechanisms. First, they enhance pyruvate production by accelerating two of the three rate-limiting steps of HK2 and pyruvate kinase (PK) as target genes for HK2 and fructose-2,6-bisphosphate in glycolysis. Second, they enhance the Warburg effect by inducing PDK1. They also phosphorylate and inactivate PDH, which reduces the conversion of pyruvate to acetyl coenzyme A, thereby allowing more pyruvate to be converted to lactate. Third, they activate LDH-5 and inhibit LDH-1, promoting the conversion of pyruvate to lactate (Feng et al., 2018; Dang et al., 2011; Clem et al., 2008).

Due to the increase of glycolysis rate, a large amount of lactate is produced, which then promotes the lactylation of histone to support the expression of c-myc (Pandkar et al., 2023). Myc regulates a series of innate and adaptive immune cells and guides their proliferation, maturation, activation and subsequent immune function events. For example, it coordinates T cell metabolic reprogramming and macrophage polarization (Wang et al., 2019; Gnanaprakasam et al., 2017; Gnanaprakasam and Wang, 2017). During immunization, the Treg-specific transcription factor, FOXP3, inhibits c-Myc signaling to reprogram Treg cell metabolism. It diverts Tregs to OXPHOS metabolism, thereby allowing Treg cells to remain active and become more adaptive in low glucose and high lactate TME (Angelin et al., 2017). In addition, Myc induces and regulates the expression of immune checkpoints, including PD-L1 (Casey et al., 2017). In turn, myc itself is regulated by other signaling pathways, such as the NF-κb pathway in B cells. If it is damaged, the myc protein of B cells cannot be upregulated after mitotic stimulation, leading to the growth defect of mature B cells (Grumont et al., 2002; Grumont et al., 2004).

## 5 Lactate affects immune cells in lymphoma

The role of lactate in immune cells in lymphoma is multifaceted and has a complex dual nature. The property of lactate allows it to both promote immune escape from tumors by affecting the function of immune cells. It also provides an energy source for certain immune cells, maintaining their function and enhancing anti-tumor immunity. In different immune cells, the effect of lactate on them is specified below.

### 5.1 Immune cells in lymphoma

**FIGURE 2:**
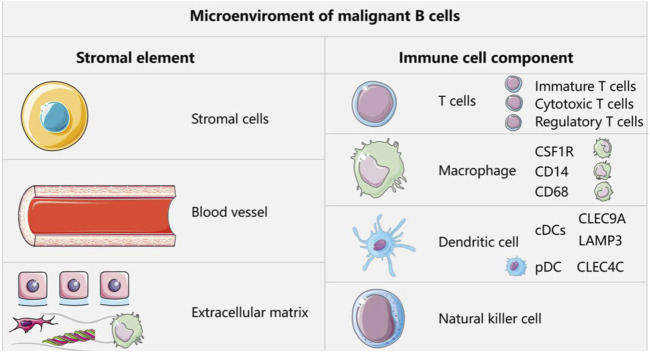
Microenvironment components of malignant B cells.

In the context of normal development, the malignant transformation of mature B cells in the germinal center or more differentiated B cells in the secondary lymphoid organs gives rise to B cell lymphoma (Scott and Gascoyne, 2014). The attack of immature B cells by exogenous antigens occurs mainly in the germinal center, a process that is tightly regulated by highly coordinated interactions between immune cells and stromal cells. The microenvironmental components of malignant B cells include a stromal component (stromal cells, vasculature, and extracellular matrix) and an immune cell component (T cells, macrophages, dendritic cells (DCs), and NK cells) (Scott and Gascoyne, 2014; Nicholas et al., 2016; Basso and Dalla-Favera, 2015). The presence of these immune cells was recently validated in a B-cell lymphoma study cohort. First, the study identified CD4 or CD8 T cell subclusters. The infiltrating T cells were classified as naïve T cells, cytotoxic T cells, regulatory T cells (Tregs), or helper T cells. The helper T cells were subsequently identified as follicular T cells (Tfh) or helper T cells 17 (Th17). In addition, six myeloid cell subclusters could be identified, including three macrophage subclusters (MPs) (CD68, CD14, and CSF1R), two conventional DC subclusters (cDCs) (CLEC9A and LAMP3), and one plasma cell-like DC subcluster (pDC) (CLEC4C) (Steen et al., 2021) (Figure 2). They affect tumor growth and behavior through their interaction with tumor cells, thereby affecting the survival rate of patients. The microenvironment of T-cell lymphoma includes multiple cell types, extracellular matrix, and soluble factors (Giudice, 1967).

### 5.2 T cells and NK cells

In the development and progression of lymphoma, the innate and adaptive immune systems work in concert. The most important effector cells are NK cells and T cells (Taylor and Gribben, 2015). Available evidence suggests that due to the heterogeneity of the immunological profile of TME, the composition of T-cell and NK-cell specific genes that characterize T-cell phenotype can effectively predict the prognosis of DLBCL patients (Autio et al., 2021).

#### 5.2.1 NK cells

**FIGURE 3:**
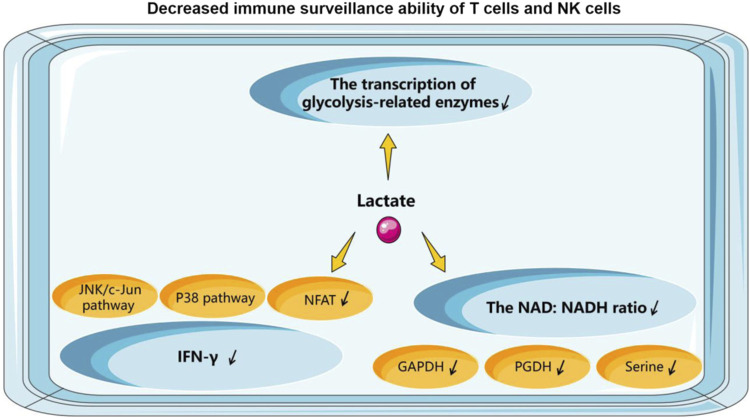
How does lactate affect the function of T cells and NK cells.

The accumulation of lactate in the lymphoma microenvironment creates an acidic environment that is an effective inhibitor of T and NK cell function and survival. Some mouse studies have confirmed that tumors with reduced lactate production develop significantly slower compared to control tumors. The pathophysiological concentration of lactate prevents the upregulation of nuclear factor of activated T cells (NFAT) in T cells and NK cells, reduces the infiltration of IFN-γ-producing T cells and NK cells (Brand et al., 2016), and diminishes the immune-surveillance role of T cells and NK cells. In B-cell lymphomas, lactate accumulation and decreased pH in the TME lead to a progressive loss of IFN-γ production by NK cells. Transfer of cells to a normal microenvironment or systemic alkalinization of lymphoma mice with oral bicarbonate restores IFN-γ expression by lymphoma-derived NK cells, and the number of NK cells in tumor-growing lymphoid organs will increase. Reactivation of NK cell-dependent IFN-γ expression can be achieved by reversing acidosis, significantly delaying tumor growth (Pötzl et al., 2017). Secondly, lactate reduces the intracellular pH of T cells, which can mechanistically affect the transcription of glycolysis-related enzymes, interfere with the rate of T cell glycolysis and proliferation, and thus reduce the activity of basic cellular metabolic pathways (Uhl et al., 2020). Finally, the NAD: NADH ratio is a key point in the metabolic control of T cells. LDH reduces nicotinamide adenine dinucleotide (NAD+) to NADH in the presence of sufficient lactate, thus generating a low NAD: NADH ratio within the cell. Lactate is depleted of the NAD-dependent enzymatic reactions of glyceraldehyde 3-phosphate dehydrogenase GAPDH and glyceraldehyde 3-phosphate dehydrogenase PGDH reactions and deprived of glucose-derived serine by this reduction stress. Eventually, lactate will be unaffected by microenvironmental acidification. It continues to impair T cell proliferation and keeps T cells in an inhibited, pro-tolerant state (Quinn et al., 2020). Metabolic profiles in EBV-infected B lymphoma cells show reduced NAD+/NADH ratios (Bonglack et al., 2021). In a study of Burkitt lymphoma, it was demonstrated that the use of LDH-specific inhibitors could result in a reduction in MYC protein levels through NAD/NADH-dependent inhibition of sirtuin-1, thereby depriving BL cells of the most important survival signal (Vettraino et al., 2013). Consequently, in lymphoma, lactate plays a pivotal role in maintaining the optimal NAD/NADH balance within the cells. The above process is shown in Figure 3.

#### 5.2.2 CD8^+^ T cells

**FIGURE 4:**
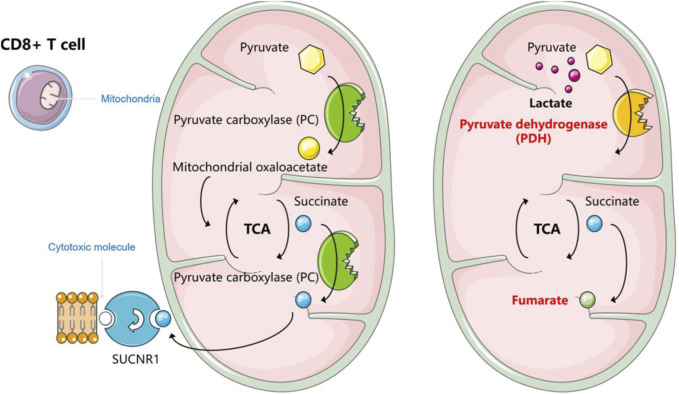
How lactate affects the function of CD8^+^ T cells.

For CD8 cytotoxic T cells, which play a central role in immune monitoring, they are cytotoxic effector cells (CTLs) (Töpfer et al., 2011). T cell receptors bind to antigenic peptides on major histocompatibility complex-like molecules (MHC) to activate T cells. Upon recognition by MHC class I molecules, activated CD8 cytotoxic T cells can efficiently destroy target cells using mechanisms such as perforin (van den Broek et al., 1996). Lymphoma employs a variety of strategies to induce CD8^+^ T cell incompetence in TME. Extracellular acidification inhibits the function of CD8^+^ T lymphocytes (Ippolito et al., 2019) (Figure 4). Mechanistically, lactic acidosis inhibits the T cell receptor-triggered JNK/c-Jun and P38 pathways. This pathway is essential for IFN-γ production, and thereby impairing the function of CD8^+^ T lymphocytes (Mendler et al., 2012). In addition to this, the anti-tumor immune response of CD8^+^ T cells is influenced by altered pyruvate utilization and succinate signaling. An experiment established an *in vitro* system in which, under normal conditions, CD8^+^ T cells rely on pyruvate carboxylase (PC) to convert pyruvate to mitochondrial oxaloacetate to replenish TCA circulating intermediates. It also shunts succinate from the TCA cycle to initiate autocrine signaling via succinate receptor 1 (SUCNR1), a proinflammatory G-protein-coupled receptor, which promotes the production of cytotoxic molecules by T cells to facilitate tumor killing. However, lactate restores the program to the traditional TCA cycle, whereby pyruvate utilization is converted from PC to PDH and succinate to fumarate (Elia et al., 2022).

However, as research continues, it has been found that lactate reveals a dual role. Lactate has also shown unusual immunoprotective effects in antitumor immunity. Lactate acts as a physiological carbon source for activated CD8^+^ T cells, as shown by *in vitro* and *ex vivo* mass spectrometry isotope tracer analysis using customized cell culture media (Kaymak et al., 2022). In addition to serving as a fuel for tumor-infiltrating CD8^+^ T lymphocytes, lactate can also induce T cell stemness and reduce apoptosis of CD8^+^ T cells during expansion through epigenetic regulation of T cell factor 7 (Tcf7), a key transcriptional regulator of T cell fate. Single-cell transcriptomic and flow cytometry analyses and *ex vivo* cultures of CD8^+^ T cells derived from mouse splenocytes and human PBMCs revealed that lactate inhibits histone deacetylase in CD8^+^ T cells and leads to increased TCF-1 expression. This transforms them into potent anti-tumor immune cells, a subpopulation of CD8^+^ T cells that express stem cell-like TCF-1 (Feng et al., 2022).

#### 5.2.3 CD4^+^ T cells

CD4^+^ T cells function mainly as paracrine, cytotoxic and regulatory agents in the immune microenvironment of lymphoma. CD4^+^ T helper cells are involved in the co-stimulation of effector lymphocytes and the activation of APCs after recognition of tumor antigens presented on MHC class 2 molecules. It enhances the ability of DCs to induce cytotoxic T lymphocyte responses, stimulates clonal expansion of activated CTLs through IL-2 secretion, and enhances macrophage and NK immune surveillance through IFN-γ production (Vettraino et al., 2013). In addition, it may have cytolytic activity itself. An animal model of B-cell lymphoma suggests that the key to the establishment of anti-TME is CD4^+^ T cells and that CD4^+^ T cells are able to predict patient prognosis (Ding et al., 2012).

**FIGURE 5:**
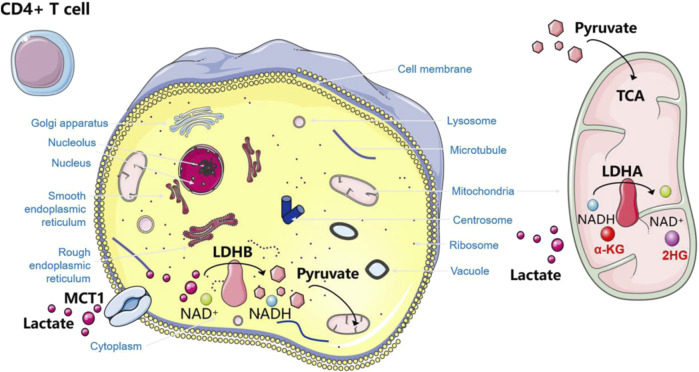
How lactate affects Th17/Treg ratio.

In CD4^+^ T cells, lactate promotes the differentiation of CD4^+^ T cells towards regulatory Treg cells to maintain their suppressive activity. It has been shown that one of the important mechanisms by which CD4^+^ T cells utilize lactate to affect the Th17/Treg ratio is an increase in the intracellular 2HG/α-KG ratio (Zhang et al., 2023) (Figure 5). CD4 + T cells take up lactate via MCT1 and accelerate the intracellular metabolism of lactate by inducing increased expression of LDHB in the cytoplasm, which catalyzes the dehydrogenation of lactate to generate pyruvate, accompanied by the conversion of NAD + to NADH. The latter enters the mitochondria and participates in the tricarboxylic acid cycle. In addition, lactate significantly increased the mitochondrial LDHA level in CD4^+^ T cells, promoting the conversion of NADH to NAD + as well as the conversion of α-KG to 2HG. Abnormally increased 2HG increases the proportion of Treg by inhibiting ATP5B-mediated mTOR phosphorylation and HIF-1α synthesis, resulting in insufficient ubiquitination and degradation together with Foxp3 (Zhang et al., 2023). Tregs have flexible metabolic patterns. Tregs can use metabolites from TME (such as lactate) as alternative energy substances to maintain their inhibitory ability in harsh environments (Watson et al., 2021). In a low glucose environment with high glycolysis in MYC amplified tumors, Tregs actively absorb lactate through MCT1, promoting NFAT1 translocation into the nucleus, thereby upregulating Programmed cell death-1 (PD-1) expression, while PD-1 expression in effector T cells is inhibited (Kumagai et al., 2022). In addition, in low glucose, lactate-rich environment, the Treg transcription factor Foxp3 reprograms T cell metabolism by inhibiting Myc and glycolysis, enhancing OXPHOS, and nicotinamide adenine dinucleotide oxidation, giving Tregs a metabolic advantage. The addition of lactate to Tregs resulted in an increase in OCR and a decrease in ECAR, which once again proves that lactate triggers stronger OXPHOS. This powerful ability to oxidize exogenous lactate greatly improves the survival rate of Tregs in TME (Angelin et al., 2017). Finally, the regulation of Tregs production relies on the mechanism of lactate acetylation through the Lys72 site in MOESIN. By transforming growth factors (TGF) - β enhances TGF in Treg cells- β Signal transduction, thereby improving their interaction with downstream SMAD3 signal transduction (Gu et al., 2022). Finally, lactate improved the differentiation of Tregs and immature T cells through acetylation, increased the expression of FOXP3, and enhanced the function of Tregs inhibititory effect on T cell proliferation. Therefore, lactate can be identified as an essential small molecule for Tregs to inhibit tumor immunity.

#### 5.2.4 Treg cells

Tregs are highly immunosuppressive CD4 T cells. Tregs are a double-edged sword in regulating immunity, either protecting immune homeostasis or suppressing immune responses (Vignali et al., 2008). Higher levels of Tregs may be associated with better outcomes in follicular lymphoma (FL), germinal center-like DLBCL, and classical Hodgkin’s lymphoma, but they have a negative prognostic impact in non-germinal center DLBCL (Tzankov et al., 2008). In the early stages of immune escape of lymphoma, Tregs can regulate the immune response, inhibit the activation of other immune cells, and maintain the stability of the immune system, which can potentially impact survival rate and immunochemotherapy resistance (Töpfer et al., 2011; Nishikawa and Sakaguchi, 2014). In B-cell non-Hodgkin’s lymphoma (FL and DLBCL), the main subtype of tumor-associated Tregs is activated Tregs. The distinguishing feature between them and resting Tregs in peripheral blood is their strong immunosuppressive ability and co-expression of checkpoint receptors (Spasevska et al., 2023). The number of Tregs in peripheral blood and tumor tissue of DLBCL patients significantly increased compared to the normal control group, and the number of Tregs significantly decreased after treatment. Tregs can enhance multiple inhibitory functions of CD8^+^ CTLs, such as granule enzyme secretion and degranulation (Su et al., 2022).

### 5.3 Macrophage

Tumor-infiltrating macrophages (also known as tumor-associated macrophages, TAM) are usually the most abundant immune cells in the TME in hematologic malignancies, including lymphomas (Franklin et al., 2014; Cassetta and Pollard, 2018). Macrophages are classified as M1/classically activated and M2/alternately activated. Based on the M1 and M2 phenotypes of TAM, M1 macrophages were found to have tumor cell killing and anti-angiogenic effects. However, M2 macrophages are preferentially located in the hypoxic zone of the tumor, which has a strong inflammatory profile (Serna et al., 2023; Cai et al., 2012), and they perform immunosuppression and promotes angiogenesis and metastasis through the expression of HIF-1α, which induces the transcription of vascular endothelial growth factor (VEGF), fibroblast growth factor (FGF), etc (Brigati et al., 2002; Marinaccio et al., 2014). In tumors, TAM is mainly composed of the M2 subtype (Mantovani et al., 2017). Various studies have demonstrated that the pro-angiogenic M2 TAM found in DLBCL (Shen et al., 2016), with increased numbers, is an independent predictor of shorter overall survival (OS) and progression-free survival (PFS) in lymphoma patients and is strongly associated with poor prognosis (Wada et al., 2012; Nam et al., 2014; Marchesi et al., 2015; Steidl et al., 2010). This depends specifically on the macrophage phenotype, either M1 (CD68/HLA-DR) or M2 (CD68/CD163) (Riihijärvi et al., 2015). Their results showed that higher CD68 expression of TAM in DLBCL patients was associated with worse outcomes (Cai et al., 2012). In conclusion, TAM plays an active role in enhancing angiogenesis in human lymphoma. It not only promotes the generation, repair, and remodeling of blood vessels and lymphatic vessels, and the growth and proliferation of lymphoma tumor cells, but also inhibits adaptive immunity, worsens patient prognosis, and enhances drug resistance (Xiong et al., 2022; Ribatti et al., 2024).

A critical factor in maintaining the tumor-promoting activity of TAM is elevated lactate levels. The mechanism by which lactate induces TAM-promoting tumor expansion is primarily through the production of VEGF and the promotion of TAM polarization to an M2-like phenotypic tumor-promoting state (Stockmann et al., 2008). In M1 TAM, lactate inhibits its function by decreasing the expression of IL-6, iNOS, and CCL2 (Certo et al., 2021). For M2 TAM, the expression of 2 transcription factors (HIF-1α and HIF-2α) is key (Pugh and Ratcliffe, 2003; Murdoch et al., 2008). Lactate signaling induces HIF-1α-dependent polarization of macrophages and activates the lactate/HIF-1α/VEGF signaling axis, which upregulates the expression of the arginine metabolizing enzyme arginase 1 (Arg 1) to provide a substrate for cancer cell proliferation to support tumor growth and upregulates VEGF to induce neovascularization to achieve a tumor-promoting state (Chang et al., 2001; Qian and Pollard, 2010). In addition, this signaling axis also induces a variety of other genes, including Fizz1, Mgl1, and Mgl2 (Colegio et al., 2014b). Second, macrophages express G protein-coupled receptor 132 (GPR132) at high levels on their surface. When macrophages sense extracellular lactate, it induces the expression of cyclic adenosine monophosphate (cAMP) and the early inhibitory protein (ICER), which upregulates ARG1, VEGF, and HIF-1α, and can also lead to M2 macrophage polarization (Chen et al., 2017). Finally, lactate actively downregulates Atp6v0d2 expression in TAM through mTOR-dependent inhibition of TFEB, a transcriptional regulator of lysosomal proteins. This inhibition mediates lysosomal degradation of HIF-2α in macrophages, and maintenance of HIF-2α leads to enhanced tumor vascularization and growth (Liu et al., 2019).

Another recent study found that lactate can also be HIF1α-independent under prolonged hypoxic conditions. NDRG3 is spared from degradation in a PHD2/VHL-dependent manner by binding to lactate, similar to HIF1α under normoxic conditions. This leads to increased NDRG3 and activation of the RAF-ERK pathway (Certo et al., 2021). Moreover, the B-cell junction of PI3K (BCAP) exacerbates the above process by promoting the reparative transformation of macrophages through histone lactonylation. It controls the pathophysiological responses associated with hypoxia, including inflammation and angiogenesis in turn (Irizarry-Caro et al., 2020; Chen et al., 2021).

Lactate-derived histone lysine lactylation (Kla) has a regulatory role in gene expression in macrophages. Kla levels are elevated when increasing concentrations of lactate treat the model system of bacterial-exposed M1 macrophages. That is, in the aerobic glycolysis that occurs during polarization of M1-type macrophages, lactate initiates a “lactate clock” by mediating the modification of the lysine at position 18 of histone H3 (Zhang et al., 2019; Latham et al., 2012). This modification drives the expression of M2-like genes during the late phase of M1-type macrophage polarization. ARG1 expression is also supported at these later stages. In addition, histone acetylation levels in the TME are associated with the production of M2 macrophage-related cancer genes (Zhang et al., 2019).

### 5.4 Dendritic cells

DCs are activated after phagocytosis and processing of antigens and are potent stimulators of the immune response against foreign antigens (Austyn, 1998). They upregulate MHC, co-stimulatory molecules, and adhesion molecules during effective antigen presentation and can stimulate naïve T cells. However, the role of DCs in lymphomagenesis is not clear, though their presence is indispensable. DCs influence tumor growth through interactions with tumor cells and T lymphocytes. Recent evidence suggests that in malignant hematologic diseases, DC loads tumor-specific antigens and generates specific anti-tumor T-cell responses (Davison, 2010). One study found that in NHL, the expression of CD62L and CCR7 (receptors essential for homing lymph nodes) was significantly reduced, leading to phenotypic alterations reduced numbers of DCs, and loss of tumor control (Fiore et al., 2006).

However, several studies have found that DCs also promotes local T-cell tolerance and an inflammatory environment. Higher ECOG scores and poorer outcomes in lymphoma patients have been associated with CD14^+^ DCs in tumor tissue (Gršković et al., 2022). Furthermore, in the Eμ-Myc model, the C/EBPβ transcription factor controls the expression of lymphoma-associated cytokines and leads to the suppression of T-cell responses to lymphoma by DCs (Rehm et al., 2014). Another study found that mice lacking the co-stimulatory receptor CD137, which is involved in the crosstalk between DCs and germinal center B cells, have a strong susceptibility to germinal center B-cell lymphomas (Middendorp et al., 2009). Finally, in T-cell lymphocyte proliferation, monocyte-derived cells fail to mature into DCs and protect tumor cells by preventing their death due to tumor IL-10 secretion (Wilcox et al., 2009). These findings demonstrate that the absence of DCs delays the progression of lymphoma.

High concentrations of lactate in the TME have been shown to contribute to the maturation and differentiation of DCs, enhance co-stimulatory molecule expression, and improve the uptake, processing, and presentation of antigens (Vermeulen et al., 2004; Tong et al., 2011). In contrast to this positive effect, most studies prefer an inhibitory effect.

First, lactic acidosis can both delay monocyte differentiation into DCs (Certo et al., 2021), impeding DC maturation, and induce monocyte differentiation into DCs with an immunosuppressive phenotype during antigen-specific autologous T cell stimulation (Nasi et al., 2013; Sutherland, 1988; Erra Díaz et al., 2020). In both allogeneic and autologous experimental settings, it was observed that lactate altered the antigen expression of DCs (immature/mature DCs, Langerhans cells) and strongly inhibited antigen presentation. In addition, a significant reduction in interleukin 12 (IL-12) secretion by DCs was found in both TADCs in MCTS co-cultures and controls supplemented with lactate during activation (Gottfried et al., 2006). In mouse gliomas, the glycolysis inhibitor diclofenac treated the inability of DCs to produce IL-12 in response to Toll-like receptor stimulation *in vitro* (Chirasani et al., 2013).

Secondly, Brown et al. reported that lactate activates G protein-coupled receptor 81 (GPR81, also known as hydroxycarboxylic acid receptor 1 or HCAR1) in DCs, which inhibits cell-surface presentation of MHCII, exerts a paracrine effect and prevents the presentation of tumor-specific antigens to other immune cells (Brown and Ganapathy, 2020). Raychaudhuri et al. found that lactate mediates the production of IL-12 through the intracellular GPR81 receptor on the surface of plasma cell-like dendritic cells (pDC). Or, the activation of calmodulin phosphatase signaling is triggered directly by cytoplasmic input from pDC via cell surface MCT proteins. Ultimately, it leads to an increase in free cytoplasmic calcium ions. It can inhibit pDC activation and type I IFN production, and affect the cellular metabolism required for effective pDC activation, leading to a tolerant phenotype (Vignali et al., 2008).

Finally, lactate contributes to the induction of Foxp3+ CD4^+^ Tregs, the major immunosuppressive cell subset in the TME, by enhancing the metabolism of tryptophan and the production of kynurenine in pDC (Raychaudhuri et al., 2019). In conclusion, in DC, excessive lactate levels in the TME impair induced monocyte differentiation and inhibit DC activation, cytokine production, and initiation of T cells.

### 5.5 Myeloid-derived suppressor cells

Myeloid-derived suppressor cells (MDSC) are a diverse group of immature myeloid cells. In addition to Tregs, MDSCs are another cell population that promotes an immunosuppressive microenvironment (De Veirman et al., 2014; Rodriguez et al., 2010; Almand et al., 2000) and plays a role in tumor induction and progression as well as immune evasion. In different lymphoma subtypes, an increase in the number of MDSCs leads to cancer progression and is associated with poor clinical outcomes (Betsch et al., 2018). This immunosuppressive effect was found to be associated with MDSC secretion of T-promoting cytokines and chemokines in mouse lymphoma studies (Schlecker et al., 2012). In addition, MDSC may play an important role in tumor tolerance as T-specific tolerogenic antigen-presenting cells (APCs) (Serafini et al., 2008).

Lactate in TME in the form of histone H3K18 lactylation modification promotes the expression of RNA methyltransferase METTL3 in tumor-infiltrating myeloid cells (TIMs). In addition, the zinc finger structural domain of METTL3 is lactonated. These effects promote mRNA methylation of Jak1 and enhance activation of the JAK1-STAT3 signaling pathway by interacting with YTHDF1 to increase translation efficiency. It promotes the immunosuppressive function of TIMs and mediates tumor immune escape (Xiong et al., 2022).

In NHL, MDSCs are regulated by NK cells. MDSCs express NKG2D ligands and activates NK cells to produce large amounts of IFN-γ (Sato et al., 2015; Nausch et al., 2008). This cell subset is inversely proportional to the number of NK cells and increases with NK cell depletion. Lactate inhibits NK cell function and increases MDSC, which can contribute even further to the inhibitory microenvironment (Husain et al., 2013).

## 6 Lactate affects immune checkpoints

### 6.1 PD-1

PD-1 is a surface inhibitory receptor expressed by macrophages, DCs, and T cells (Freeman et al., 2000). PD-1 binds to PD-L1 (expressed on the surface of APCs) and inhibits T-cell cytokine production and cell-cycle progression (Keir et al., 2008). The prevalence of PD-1 expression in DLBCL ranges from 39.5% to 68.6% and is usually increased on tumor-infiltrating T cells (Yamamoto et al., 2008), with higher levels being associated with poorer prognosis (Zhang et al., 2015; Zhang et al., 2016). PD-1 blockade has shown promise in phase 1 trials in DLBCL (Xu-Monette et al., 2018). Furthermore, numerous experiments have shown that PD-L1 is similarly highly expressed in lymphoma cells (Li et al., 2018). The DLBCL subgroup with PD-L1 is associated with a poor prognosis compared to the PD-1-negative subgroup (Laurent et al., 2015; Kiyasu et al., 2015).

It has been shown that M2-like macrophages, Treg cells, and certain inhibitory molecules (e.g., PD-L1) can be involved in mediating HIF1α-VEGF signaling pathway activity, leading to more active lactate metabolism (De Saedeleer et al., 2012; Seth et al., 2017; Vaupel and Multhoff, 2016). Conversely, lactate can also impact PD-1 expression. Lactate regulates the active checkpoint of Treg cell function in TME by upregulating PD-1 expression. As shown previously, Treg cells acquire higher PD-1 expression than effector T cells in highly glycolytic tumors. This mechanism means that lactate can upregulate PD-1 expression to enhance Treg cell function in TME and inhibit effector T cell activity, which promotes immune escape from lymphoma (Kumagai et al., 2022). Similarly, Feng et al. reported that tumor cell-derived lactate mediates the upregulation of PD-L1 through activation of its receptor GPR81, which is dependent on LDHA, and this in turn regulates macrophage polarization and allows tumor cells to evade cytotoxic T-cell targeting (Feng et al., 2017).

### 6.2 Cytotoxic T-lymphocyte-associated protein 4 (CTLA-4)

CTLA-4 is an inhibitory surface receptor with significantly elevated expression in lymphoma tissues and is an indicator for the early diagnosis and clinical treatment of lymphoma. It increases the proportion of lymphoma stem cells and induces the proliferation of Treg cells through the TGF-β pathway, which promotes the growth of lymphoma and recruits more immunosuppressive cells. CTLA-4 inhibits anti-tumor immune response and is closely related to the malignancy of lymphoma (Chen et al., 2021). Lactate promotes CTLA-4 expression in a Foxp3-dependent manner and promotes ubiquitin-specific peptidase 39 (USP39)-mediated RNA splicing in tumor-infiltrating Treg cells. This lactate-Foxp3-USP39-CTLA-4 signaling axis mediates high expression in tumor-infiltrating Treg cells in order to maintain Treg cell immunosuppressive function (Ding et al., 2024).

### 6.3 V-domain ig suppressor of T-cell activation (VISTA)

VISTA is a macrophage-negative immune checkpoint regulator that is highly expressed in tumor-infiltrating myeloid cells. In T-cell lymphomas, VISTA expression (88.1%) was found predominantly in CD68^+^ macrophages, and it was much higher than the expression of PD-L1 (68.7%) (He et al., 2021; Murga-Zamalloa et al., 2020). In a study of B-cell lymphoma, VISTA was found on the surface of monocytes from all patients. Its activation drove macrophages towards an M2-like pro-tumorigenic phenotype and promoted cancer cell phagocytosis. It also reduces the antigen-presenting ability of T cells at acidic pH (Lin et al., 2024), and mediates the binding of multiple histidine residues along the edge of the extracellular structural domain of VISTA to the adhesion and co-inhibitory receptor P-selectin glycoprotein ligand-1 (PSGL-1), which selectively participates in the inhibition of T cell proliferation and cytokine production. The development of acidic pH-selective antibodies against VISTA or its receptor PSGL-1 has been shown to reverse immunosuppression *in vivo* (Johnston et al., 2019). Considering that lactate can influence the pH of TME (Volk et al., 1993), it may affect the immune content associated with VISTA.

## 7 Important signaling pathways involved in lactate mediated immune regulation

### 7.1 mTOR signaling pathway

Activation of the PI3K/AKT/mTOR pathway occurs in lymphoma and is associated with p53, HIF-1α, and MYC (Sander et al., 2012; Argyriou et al., 2011; Wong et al., 2010; Rasul et al., 2012). A study found that mice expressing active Akt in lymphocytes progressively develop autoimmunity and lymphoma (Rathmell et al., 2003). mTOR is a ubiquitously expressed and highly conserved serine/threonine kinase downstream of PI3K/AKT. Transcription of several metabolic genes requires the involvement of mTOR complexes 1 and 2 (mTORC1 and 2) activation (Inoki et al., 2005). Sustained activation of mTORC1 occurs in a large number of hematopoietic and non-hematopoietic malignancies (Sabatini, 2006). It controls of a range of metabolic processes including glycolysis and mitochondrial metabolism (Laplante and Sabatini, 2013; Guertin and Sabatini, 2007). Therefore, aberrant activation of the PI3K/AKT/mTOR signaling pathway leads to enhanced metabolic activity in NHL (Blachly and Baiocchi, 2014). Lactate concentration can reflect the activation of mTOR signaling pathway in B-cell lymphoma (Lee et al., 2013). Increased lactate can result in reduced glucose consumption, upregulation of mitochondrial respiratory genes, and inhibition of mTORC1 activity (Erra Díaz et al., 2020). It promotes the differentiation of monocyte-derived DCs.

Following lactate efflux, mTORC1 is inhibited, which inhibits the production of pro-inflammatory cytokines and the cytotoxic activity of T cells (Balgi et al., 2011). Extracellular acidosis inhibits the mTOR signaling pathway and downregulates protein synthesis (Balgi et al., 2011; Pouysségur et al., 1982) thereby impairing immune cell function. For example, impaired NKT cell function leads to a reduction in effector factors (Xie and Bai, 2016), interfering with the antitumor effects of natural NK.

### 7.2 NF-κb signaling pathway

Changes in lactate metabolism can be associated with dysregulation of NF-κB signaling (Kooshki et al., 2022). Lactate flows into endothelial cells through MCT1 to induce the activation of NF-κB and support the drive of tumor angiogenesis (Scott and Gascoyne, 2014). The majority of B-cell lymphomas are known to activate constitutive phenotypes of the NF-κB pathway, which in turn promotes sustained lymphocyte proliferation and survival. Recent studies have confirmed that NF-κB is expressed in DLBCL and correlates with poor prognosis. It is considered to be an important pathogenic factor and one of the major therapeutic targets in lymphoma (Scott and Gascoyne, 2014; Zhang et al., 2016; Jost and Ruland, 2007; Davis et al., 2001; Yu et al., 2017; Ben-Neriah and Karin, 2011).

NF-κB signaling pathway is involved in the regulation of lactate on immune cells. Lactate can regulate macrophage phenotype through its receptor GPR81-mediated AMPK/LATS activation, which inhibits lipopolysaccharide (LPS)-stimulated NF-κB (Yang et al., 2020). Furthermore, Puig-Kroger et al. demonstrated that lactate impairs the maturation of MDDC phenotype and function induced by LPS, which is mediated by the inhibition of NF-κB activation. Furthermore, the pathway has been demonstrated to reduce the production of inflammatory cytokines (CCL2 and TNFα) in monocytes (Jost and Ruland, 2007; Peter et al., 2015; Dietl et al., 2010).

## 8 Novel therapeutic approaches targeting lactate metabolism

In the treatment of lymphoma, lactate metabolism-targeted drugs, such as LDH inhibitors, are currently being developed. Cancer cells are enthusiastic about converting excessive glucose into lactate through LDH absorption. LDH inhibitors can effectively block glycolysis and ATP production, directly affecting tumor growth and progression and reducing tumor acidity. Inhibition of LDHA activity reduces lactate production and helps reduce lactate accumulation in the TME, thus potentially reducing the acidic burden in the TME and improving immune cell function. In the latest mouse experiment, it was found that inhibiting LDH can effectively suppress aerobic glycolysis and reprogram T-cell metabolism. Due to the permanent deficiency of LDHA, the development of effector CD8^+^ T cells with strong anti-tumor activity is hindered (Hermans et al., 2020). Inhibition of LDHA by siRNA or by a small molecule inhibitor (FX11) was found in one study to reduce ATP levels and induce significant oxidative stress and cell death. When combined with the NAD synthesis inhibitor FK866, FX11 induced lymphoma regression. Thus, LDHA inhibition by FX11 represents a promising and tolerable treatment for LDHA-dependent tumors (Le et al., 2010). The ubiquitin ligase F-box and WD repeat domain contains 7 (Fbw7) targeting various oncogenic proteins for protein hydrolysis. It may support future ABC-DLBCL therapy by targeting LDHA-related inhibition, such as aerobic glycolysis reprogramming, in DLBCL (Yao et al., 2022).

In addition, the expression of MCT promotes lactate export. In one study, AZD3965-mediated disruption of MCT1 activity was found to result in suppression of NHL cell viability and extracellular lactate accumulation, along with increased apoptotic cell death. It suggests that MCT1 could be a target for the treatment of non-Hodgkin’s lymphoma (DLBCL) with high expression of MCT1/low expression of MCT4 (Afonso et al., 2019).

However, lactate-targeted monotherapy has limited therapeutic efficacy due to its off-target effect. Therefore, combining lactate targeting with other therapies (e.g., mTOR inhibitors, anti-PD-1/PD-L1 therapies, anti-CTLA-4 therapies) may be an alternative treatment strategy.

**FIGURE 6:**
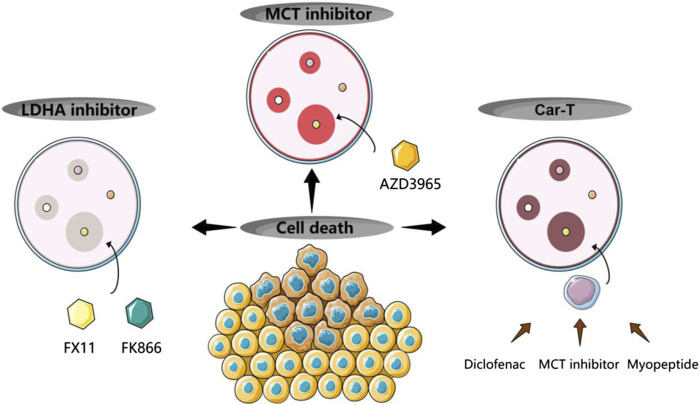
New treatments of lactate metabolism.

Advanced cell therapy (ACT) using chimeric antigen receptor (CAR)-engineered T cells is highly effective in the treatment of refractory lymphoid malignancies (Chavez et al., 2019; Zhang et al., 2020). However, only a minority of patients experience long-term remission (Schuster et al., 2019; Neelapu et al., 2017). A major obstacle to long-term cancer remission with CAR-T cell therapy is the poor persistence of CAR-T cells (Byrne et al., 2019). Although immune checkpoint inhibitor therapy has been proposed to restore exhausted T cell function, such therapy has limitations (Balar and Weber, 2017). Therefore, the combination of Car-T with targeted lactate production (inhibition of LDH) or secretion (inhibition of MCT) has great potential and value in cancer treatment. Activation of CAR genetically engineered T cells occurs in a harsh tumor environment with low pH immune suppression. There have been proposals to limit lactate secretion through a combination of targeted MCT therapy as a potential adjuvant for T cell adoptive transfer. Diclofenac can selectively inhibit tumor cell metabolism and proliferation by suppressing MCT4 (Moreno-Sánchez et al., 1999), with little effect on T cell cytokine production (Renner et al., 2019). The metabolic demands of tumor cells and activated T cells result in the production of lactate. CAR T cells express high levels of MCT1 and MCT4 upon activation. Expression of MCT proteins promotes lactate output. Pharmacological blockade of MCT1 has been shown to selectively impair B-cell tumor growth without impeding the antitumor potential of CD19-specific CAR T cells. This makes combining MCT inhibitors with CAR-T therapies a possible treatment against B-cell malignancies (Lopez et al., 2023). In addition, in adoptive immunotherapy, eliminating LDHA at the germline level produces T-cell offspring with limited anti-tumor function, and the temporal regulation of LDH activity is one of the important factors to consider (Hermans et al., 2020). Finally, recent studies have shown that myopeptides can act as a novel factor to buffer TME and improve the quality of T cells amplified *in vitro* by adoptive immunotherapy (Tu et al., 2021) (Figure 6).

## 9 Conclusion

Tumor cells and other cell types in the microenvironment not only compete for nutrients but also simultaneously produce immunosuppressive metabolites (e.g., lactate) that lead to immune escape (Ngwa et al., 2019). Lactate is mainly produced within the TME by cells that utilize aerobic glycolysis (Warburg metabolism). Otto Warburg was one of the first scientists to identify lactate as a characteristic product released by tumor cells. Tumor tissues contain significantly higher levels of lactate than normal tissues: the immune response changes in response to significant alterations in tissue metabolism. Immune cells can sense various signaling changes in the TME and produce specific immune functions in response to these stimuli. Meanwhile, higher lactate levels are strongly associated with a poorer prognosis in cancer patients. Increasing evidence suggests that lactate in the TME plays an important role in the regulation of metabolic pathways, immune responses, and cellular signaling, and severely affects tumor growth, progression, drug resistance, and even epigenetics.

Due to its physiological background of activation, T cells may be an important determinant of overall therapeutic efficacy. Tumor cells and activated T cells produce lactate at a high rate, leading to acidic and hypoxic TME and producing immunosuppressive effects. More seriously, there is no bottleneck for lactate accumulation in activated T cells. And research has found that MCT-1 is involved in this process. In addition, the dynamic regulation of MCT1 and MCT4 is inhibited in acidic environments. When the MCT subtype is inactivated, H ions and lactate accumulate inside the cell, ultimately inhibiting impaired glycolysis. Maintaining an acidic state may trigger an increase in the threshold of autoimmune response (Wu et al., 2020). Therefore, the combination of CAR-T cell therapy and acidic immunosuppressive therapy in TME has obvious advantages and deserves attention and further discussion. However, one of the current challenges is that T cells and tumor cells share many common metabolic characteristics, such as compounds that inhibit MCT subtypes that may be shown to have antagonistic effects on T cell-mediated responses. To improve the limitations of its therapeutic effect, in addition to improving the persistence of T cell effects after adoptive transfer, the enhancement of anti-tumor function after infusion is also a topic that we need to continue exploring.

In this context, the combination of targeted lactate metabolism and immunotherapy represents a promising therapeutic approach for lymphoma. Consequently, it is essential to elucidate the regulation of lactate and its metabolism on the metabolic profile of immune cell function in TME. This is not only important for improving the prognosis of immunotherapy but also provides useful evidence for the choice of therapeutic strategies. However, further preclinical studies are still needed to explore the potential of lactate metabolism as a therapeutic target, which is a future challenge for lactate-targeted therapy.
